# Enhancing and optimization for efficient removal of nickel ions by modified activated carbon

**DOI:** 10.1038/s41598-026-46490-1

**Published:** 2026-04-17

**Authors:** Shimaa M. Abdel-Moniem, Reem Mohammed, Hanan S. Ibrahim, Mohamed Eid M. Ali

**Affiliations:** 1 Water Pollution Research Department, Institute of Environment and Climate Changes Research, National Research Centre, El-Buhouth St., Dokki, Cairo, 12622 Egypt; 2 Physics Department, Faculty of Science, Aim Shams University, Abbassia, Cairo, 11566 Egypt

**Keywords:** Modified activated carbon, Nickel ions, Response surface methodology, Optimal removal capacity, Chemistry, Engineering, Environmental sciences, Materials science

## Abstract

This study aims to enhance the adsorption capacity of activated carbon (AC) and optimize the cumulative effect of various operational parameters on the removal of Ni (II) ions, providing a comprehensive understanding of the removal process. Activated carbon was modified using the Fenton oxidation process to produce modified activated carbon (MAC). The functional groups and morphological features of MAC were characterized using FTIR spectroscopy, particle size analysis, high-resolution scanning electron microscopy (HRSEM), and surface area measurements. To optimize the removal efficiency, response surface methodology (RSM) was employed using a central composite design (CCD). The predicted removal capacities showed good agreement with experimental values, with a determination coefficient (R^2^) of 0.92. The maximum removal capacity achieved was 210 mg/g under optimal conditions: 150 min contact time, pH 1, a low adsorbent dose of 0.2 g/L, and initial Ni (II) concentration of 125 mg/L. The adsorption isotherm analysis indicates that the Langmuir, Freundlich, and Dubinin–Radushkevich (D–R) models adequately describe the adsorption of Ni(II) onto MAC. The D–R model results further suggest that the adsorption process is mainly controlled by chemisorption, potentially through ion-exchange mechanisms. These findings demonstrate that MAC, along with the proposed predictive models, offers a promising approach for optimizing Ni (II) removal efficiency in wastewater treatment applications

## Introduction

Rapid urbanization and accelerated industrial activities have significantly contributed to the degradation of water quality through continuous discharging of wastewater that contains toxic heavy metals into aquatic ecosystems^1–4^. Among environmental pollutants, heavy metals have attracted considerable attention due to their non-biodegradable nature, long-term persistence, and severe toxicological impacts on both ecological systems and human health^4–7^. Nickel (Ni (II)) ions are concidered one of the most prevalent and hazardous heavy metals found in industrial effluents, and their presence in aquatic environments poses a significant threat due to their carcinogenic, mutagenic, and bioaccumulative properties. Major anthropogenic sources of nickel contamination include electroplating, battery manufacturing, galvanization, textile dyeing, alloy production, smelting, mining, and metal surface finishing industries^8–11^. Once released into water bodies, Ni (II) ions can persist and accumulate in sediments and biota, entering the food chain and resulting in potential risks to human and environmental health. Therefore, developing efficient, sustainable, and cost-effective technologies for the removal of nickel ions from contaminated water is a pressing environmental priority^12,13^. A wide range of conventional methods has been applied for the remediation of heavy metal-contaminated wastewater, including chemical precipitation, membrane filtration, ion exchange, and electrochemical techniques^14–16^. However, these conventional approaches are often associated with several limitations such as high operational costs, low removal efficiencies at trace concentrations, and the generation of toxic secondary waste or sludge^17–20^. Among the various decontamination strategies, adsorption has emerged as one of the most effective, economical, and scalable technologies for heavy metal removal from aqueous media^21–23^. In particular, modified activated carbon (MAC) has gained attention due to its high surface area, tunable surface functionalities, and strong binding affinity for metal ions.

Despite prior reports on Fenton-modified carbons, existing studies rarely address the combined aspects of process optimization, regeneration efficiency, and sustainability, which are crucial for practical wastewater treatment applications ^24,25^. Moreover, the systematic investigation of operational parameters such as pH, contact time, adsorbent dosage, and initial Ni (II) concentration and mechanistic understanding of Ni (II) adsorption remains limited^26–29^. The central composite design (CCD), a commonly used design in RSM, facilitates the construction of accurate predictive models and reduces experimental efforts by determining significant variables and their interactions efficiently ^30,31^.

To address these gaps, the present study aims to develop a Fenton-modified activated carbon (MAC) with high adsorption capacity for Ni (II) ions and to optimize the adsorption process using Response Surface Methodology (RSM) based on a central composite design. The study further investigates the adsorption mechanism through surface characterization and isotherm modeling. In addition, the recyclability and regeneration efficiency of MAC over multiple cycles are evaluated, confirming its potential as a practical, sustainable, and cost-effective material for efficient Ni(II) removal from wastewater  (Fig. 1).

**Figure:**
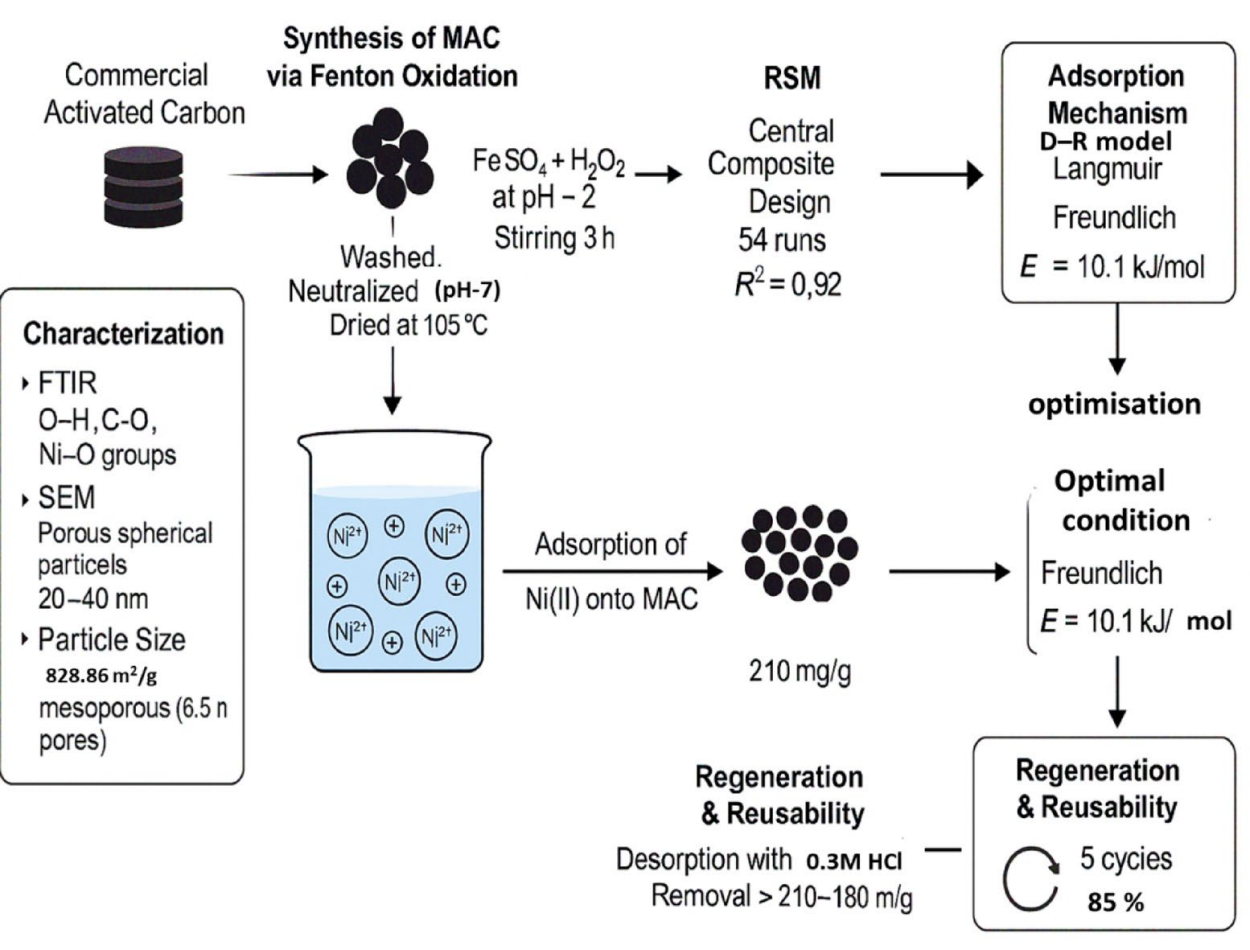
Schematic representation of the experimental workflow for Ni(II) removal using Fenton-modified activated carbon (MAC), including preparation, characterization, optimization via RSM, isotherm modeling, and regeneration studies.

## Materials and methods

### Materials

Commercial powdered activated carbon (AC) was obtained from Nasr Chemical Industries, Egypt. Hydrogen peroxide (H_2_O_2_, 35%), sulfuric acid (H_2_SO_4_, 98%), hydrochloric acid (HCl, 37%), and ferrous sulfate heptahydrate (FeSO_4_·7H_2_O) were purchased from Fisher Scientific, UK. Nickel  (II) chloride pentahydrate (NiCl_2_·5H_2_O), supplied by Merck, was used to prepare the stock solution of Ni (II) ions in deionized water. All chemicals used in this study were of analytical reagent grade and used without further purification.

### Preparation of modified activated carbon (MAC)

Modified activated carbon (MAC) was synthesized from commercial powdered activated carbon (AC) via a Fenton-based pre-oxidation process using hydrogen peroxide (H_2_O_2_) and ferrous sulfate (FeSO_4_). The Fenton modification parameters, including the Fe^2^⁺/H_2_O_2_ ratio, pH, and reaction time were selected based on both literature precedents and preliminary experiments to maximize surface functionalization while minimizing structural damage to the carbon matrix. Initially, 5 g of AC were dispersed in deionized water under continuous stirring. The suspension pH was adjusted to 2 using 1 M sulfuric acid (H_2_SO_4_), as this acidic environmental optimizes hydroxyl radical generation during the Fenton reaction and promotes the formation of oxygen-containing functional groups. Subsequently, 4 mmol of FeSO_4_ and 50 mmol of H_2_O_2_ were added to the suspension, a ratio determined to provide sufficient hydroxyl radicals for surface oxidation without excessive degradation of the carbon structure. The mixture was stirred for 3 h, which preliminary trials indicated as sufficient for achieving maximal surface functionalization.

After the reaction, the oxidized carbon was thoroughly washed with 0.1 N HCl to remove residual iron and reaction by-products, followed by repeated rinsing with deionized water until neutral pH (7) was reached. Finally, the MAC was dried at 105 °C for 24 h to eliminate residual moisture.

### Characterization of modified activated carbon

The changes in the surface functional groups of the modified activated carbon (MAC) were examined using a Fourier Transform Infrared (FTIR) spectrophotometer (Jasco-630). For FTIR analysis, pellets were prepared by thoroughly mixing 2 mg of ball-milled MAC powder with 100 mg of spectroscopic-grade potassium bromide (KBr). The infrared spectra were recorded over the wavenumber range of 4000–400 cm^−1^.

In addition, the specific surface area, pore size distribution, and total pore volume of the samples were determined using nitrogen adsorption–desorption isotherms measured by a NOVA Touch surface area and porosity analyzer (Quantachrome Instruments, USA) at 77 K. Prior to the sorptometric measurements, all samples were degassed at 423 K for 12 h to remove any moisture or volatile impurities.

The morphological features and surface texture of MAC were further investigated using scanning electron microscopy (SEM) with a Quanta 250 FEG (Field Emission Gun) microscope.

### Response surface methodology

According to the response surface methodology, the relation between the response variable $(y$) and the input variables (${{\boldsymbol{x}}}_{{\boldsymbol{i}}}$) is given by Box and Wilson^32^:

1 $$y=f\left({x}_{1},{x}_{2},{x}_{3},\dots ,{x}_{k}\right)+ \epsilon ,$$

$f$ is the response function,$k$ gives the total number of independent variables.$\epsilon$ is the statistical error that represents the effect of other sources on the response, such as measurement error and background noise. Since the function $f$ is unknown, then we need an approximate form for it. The second-degree polynomial is a widely used approximation to the true response surface $f$, which is given by:

2 $$y = \beta _{0} + \sum\nolimits_{i}^{k} {\beta _{i} x_{i} } + \sum\nolimits_{i}^{k} {\beta _{{ii}} x_{i}^{2} } + \sum\nolimits_{{i = 1}}^{k} {\sum _{{j = i + 1}}^{k} \beta _{{ij}} x_{{ij}} + \epsilon ,}$$

where, β’s are unknown constants. To estimate the values of these constants, a set of experiments were carried out where for each experiment the response ${\boldsymbol{y}}$ is measured for specific setting of the independent variables. The sum of these setting builds the response surface design or the experimental design. By substitute the measured values of the response and the corresponding independent variables into Eq. (2), we get a group of equations that can be solved using the method of least square^33,34^. The central composite design (CCD) is applied with total number of experimental runs equal 54^35,36^. Table 1 shows the complete set of the experimental design, where the five–level/four-variable CCD is implemented to investigate the influence of the four independent variables (time, pH, dose, and initial Ni (II) ions concentration) on the removal capacity. Ni(II) uptake capacities (Q_t_, mg/g), are calculated using Eq. (3):

3 $${Q}_{t}=\frac{{(\mathrm{C}}_{0}-{\mathrm{C}}_{\mathrm{f}})*\mathrm{V}}{\mathrm{M}}$$

where, C_0_ (mg/L) is the initial concentration of Ni (II), C _f_ (mg/L) is the equilibrium concentration of Ni (II) in aqueous solution, V (L) is the volume of Ni (II) ions solution, M (g) is the mass of the adsorbent, and Q_t_ (mg/g) is the calculated Ni (II) adsorption amount onto MAC through batch adsorption method.

**Table 1:** CCD of the experiment and the response results.

Run	Time (min) x_1_	pH x_2_	Dose (g/l), x_3_	Ni Conc (mg/L) x_4_	Experimental capacity (mg/g)	Predicted capacity (mg/g)
1	90	5	0.8	25	21.01	19.33
2	90	5	0.8	125	60.75	71.25
3	120	3	0.5	100	100.18	99.05
4	60	6	0.5	50	56.68	69.28
5	90	6.5	0.8	75	43.38	47.47
6	60	3	0.5	100	79.38	87.14
7	60	6	0.5	100	90.72	92.81
8	60	3	0.5	100	81.28	87.14
9	90	5	0.8	25	21.18	19.34
10	120	6	0.5	50	62.40	68.17
11	60	6	1.1	50	22.33	23.38
12	60	3	0.5	50	56.16	60.69
13	90	1	0.8	75	56.25	63.93
14	60	3	1.1	100	49.09	45.99
15	120	3	0.5	50	56.16	63.48
16	120	6	1.1	100	60.14	48.57
17	90	5	0.8	75	41.72	40.40
18	90	5	0.2	75	141.00	136.65
19	30	5	0.8	75	41.25	36.32
20	60	3	1.1	100	47.25	45.99
21	90	5	0.8	125	61.59	71.26
22	90	1	0.8	75	75.94	63.93
23	120	3	1.1	50	28.36	28.41
24	120	6	1.1	50	30.73	22.13
25	120	6	1.1	100	59.09	48.57
26	60	3	0.5	50	55.12	60.69
27	90	5	1.4	75	38.39	45.81
28	90	5	0.8	75	42.19	40.40
29	90	6.5	0.8	75	40.31	47.47
30	60	6	1.1	50	20.33	23.38
31	120	3	1.1	100	62.72	57.78
32	150	5	0.8	75	41.69	45.67
33	120	6	0.5	50	63.44	68.17
34	60	6	1.1	100	40.91	40.70
35	150	5	0.8	75	43.13	45.67
36	120	3	1.1	100	61.00	57.77
37	120	6	0.5	100	98.00	100.82
38	120	3	0.5	50	57.20	63.49
39	120	3	1.1	50	26.38	28.41
40	90	5	0.8	75	41.25	40.40
41	90	5	0.8	75	42.19	40.40
42	60	6	1.1	100	50.91	40.70
43	120	3	0.5	100	106.00	99.06
44	120	6	0.5	100	96.00	100.82
45	60	3	1.1	50	28.84	25.75
46	90	5	1.4	75	28.39	45.81
47	60	6	0.5	100	94.08	92.82
48	60	3	1.1	50	26.98	25.75
49	90	5	0.2	75	142.50	136.66
50	60	6	0.5	50	62.40	69.29
51	90	5	0.8	75	43.13	40.40
52	90	5	0.8	75	42.19	40.40
53	30	5	0.8	75	41.25	36.32
54	120	6	1.1	50	30.73	22.13

Batch adsorption experiments were conducted to determine the optimum conditions for Ni (II) removal. The mixtures were agitated using a mechanical shaker at 150 rpm. After reaching equilibrium, the solutions were filtered using Whatman® No. 41 filter papers to separate the solid adsorbent. Ni (II) ion concentrations in the filtrates were measured using inductively coupled plasma optical emission spectrometry (ICP-OES, Agilent 5100, Australia), following the standard methods for the examination of water and wastewater^37^. All experiments were performed in triplicate, and the results are reported as mean values. The relative standard deviation (RSD) for the triplicate measurements was maintained below 1%, indicating good repeatability and reliability of the data.

The experimental variables listed in Table 1 were directly input into a MATLAB script to solve the second-order polynomial equation (Eq. 2) derived from the response surface methodology (RSM) model. To avoid the chemical precipitation of nickel ions as insoluble hydroxide species, the pH values used in the experimental design were restricted to values not exceeding 6.5. This precaution is based on the Pourbaix diagram of nickel, which indicates that at pH values ≥ 7.5, Ni (II) ions are likely to precipitate as various nickel hydroxide species^38,39^. Therefore, maintaining the pH below 6.5 ensures that Ni (II) remains in a soluble ionic form, allowing accurate evaluation of its adsorption onto the modified activated carbon.

### Kinetic modeling

Time-dependent adsorption data were analyzed using the pseudo-first-order (PFO) and pseudo-second-order (PSO) kinetic models^40,41^, based on their linearized forms shown in Eqs. (4) and (5). Linear regression was applied to determine the kinetic parameters and to evaluate the goodness-of-fit (R^2^) for each model.

4 $$\mathrm{ln}\left({\mathrm{q}}_{\mathrm{e}}-{\mathrm{q}}_{\mathrm{t}}\right)=\text{ ln}{\mathrm{q}}_{\mathrm{e}}-{\mathrm{K}}_{1}\text{ t}$$

5 $$\frac{\mathrm{t}}{{\mathrm{q}}_{\mathrm{t}}}=\frac{\mathrm{t}}{{\mathrm{q}}_{\mathrm{e}}}+\frac{1}{{\mathrm{k}}_{2}{{\mathrm{q}}_{\mathrm{e}}}^{2}}$$

where k_1_ (min^−1^) and k_2_ (g mg^−1^ min^−1^), represent the rate constants of the PFO and PSO kinetic models, respectively.

### Adsorption isotherms

To evaluate the adsorption behavior of Ni (II) ions onto the modified activated carbon (MAC), a series of isotherm studies were conducted by varying the initial Ni (II) concentrations from 10 to 100 mg/L. During these experiments, other independent variables, including MAC dosage, contact time, and pH were maintained at their previously optimized values, as determined from earlier experiments assessing the influence of each parameter on the adsorption performance. Three commonly used adsorption isotherm models; Langmuir, Freundlich, and Dubinin–Radushkevich (D–R) were applied to analyze the experimental data and to elucidate the adsorption mechanism^42,43^. These models help in determining whether the adsorption process occurs on a homogeneous or heterogeneous surface, as well as providing insights into the nature and energy of the adsorption interaction.

### Recyclability studies

The recyclability of the prepared modified activated carbon (MAC) and commercial powdered charcoal was evaluated to assess their reusability in the adsorption of Ni (II) ions. After the first adsorption cycle, the spent adsorbents were separated from the solution via filtration and thoroughly washed with distilled water, followed by desorption using diluted hydrochloric acid (HCl) at varying concentrations (0.1, 0.2, 0.3, and 0.4 M) to remove the adsorbed nickel ions. Subsequently, the adsorbents were air-dried at room temperature and reused in subsequent adsorption cycles. This adsorption–desorption–regeneration process was repeated four times to complete a total of five consecutive adsorption cycles. The regeneration efficiency (RE %) of each adsorbent was calculated using Eq. 6^44^:

6 $$RE \%={\frac{{q}_{r}}{q}}_{0}x 100$$

where, ${q}_{r}$ and ${q}_{0}$ denote the adsorption capacity (mg/g) after regeneration and before regeneration (original capacity) (mg/ g), respectively.

## Results and discussion

### Characterization of MAC

**Fig. 2:**
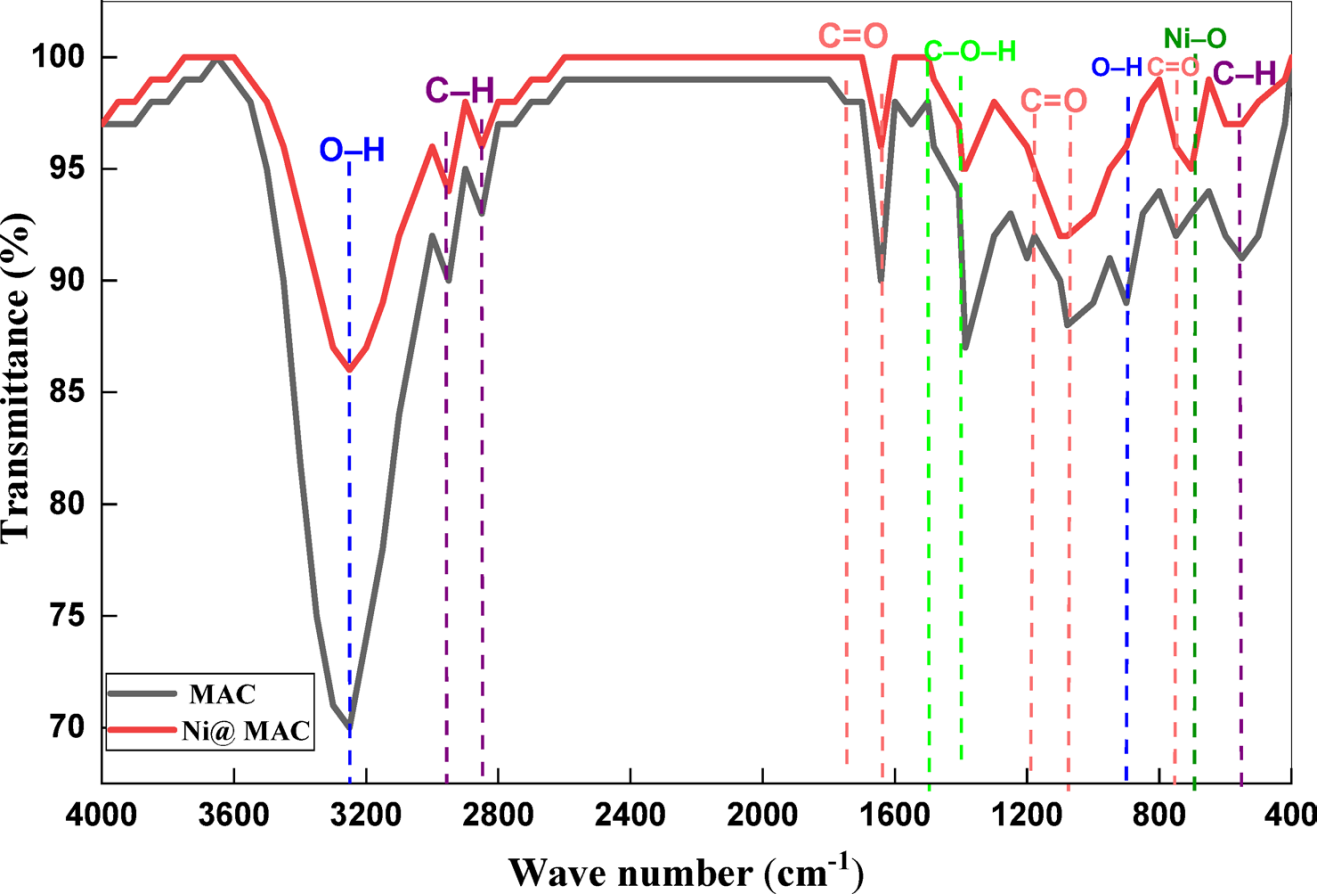
FTIR spectra of the MAC before and after Ni (II) adsorption.

Figure 2 presents the FTIR spectra of modified activated carbon (MAC) before and after Ni (II) adsorption, providing insights into the surface functional groups which are involved in the adsorption process. The spectrum of pre-oxidized MAC exhibits a broad and intense absorption band in the range of 3000–3600 cm^−1^, corresponding to the O–H stretching vibrations of hydroxyl groups, associated with phenolic and carboxylic functionalities. Moderate bands observed at 2950 cm^−1^ and 2851 cm^−1^ are attributed to aliphatic C–H stretching vibrations. Prominent peaks at 1750 cm⁻^1^ and 1642 cm^−1^ indicate the stretching vibrations of carbonyl (C = O) groups, which are essential for metal ion complexation. Additionally, weak to moderate peaks at 1482 cm^−1^, 1407 cm^−1^, and 1387 cm^−1^ correspond to C–O–H bending and in-plane bending of O–H groups. Bands at 1202 cm^−1^ is assigned to out-of-plane bending modes of C = O, while the peak at 1078 cm^−1^ is related to C–O stretching vibrations. Other notable bands appear at 899 cm^−1^, 747 cm^−1^, and 554 cm^−1^, which can be assigned to out-of-plane bending of O–H, C = O, and C–H functional groups, respectively.

Following Ni (II) adsorption, significant spectral changes are observed. A general decrease in the intensity of characteristic bands, particularly those related to O–H and C = O stretching, suggests the participation of these functional groups in the adsorption process. Notably, new peaks emerged at 573 cm^−1^ which are assigned to Ni–O vibration, confirming the binding of nickel ions to oxygen-containing groups on the MAC surface. Additionally, band 643 cm^−1^ are attributed to Ni–COO vibration, indicating chemical interactions between Ni (II) ions and carboxyl functional groups. These spectral modifications collectively affirm the involvement of surface functional groups, particularly hydroxyl and carbonyl moieties, in the complexation and adsorption of Ni (II) ions onto the MAC surface ^39,45^.

**Fig. 3:**
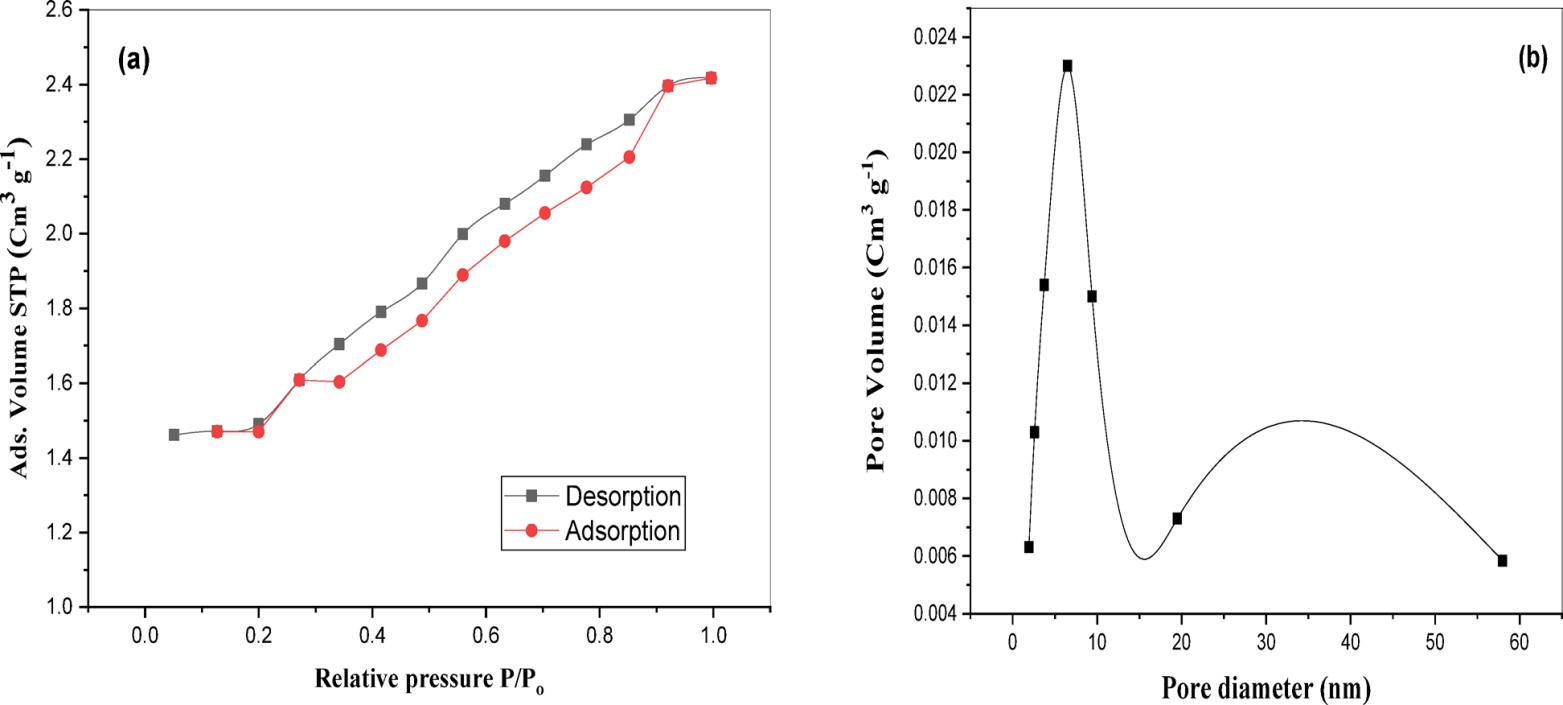
(**a**) Nitrogen adsorption and desorption isotherm of the MAC, (**b**) The pore size distribution of the MAC.

The surface textural properties of the modified activated carbon (MAC) were investigated using nitrogen adsorption–desorption isotherms at 77 K. The corresponding isotherm is shown in Fig. 3a, exhibiting a Type IV isotherm with a noticeable H_3_-type hysteresis loop, which is indicative of mesoporous structures, as per the IUPAC classification. The adsorption and desorption branches clearly diverge, suggesting the presence of capillary condensation phenomena typical in slit-shaped mesoporous.

The calculated BET surface area of the MAC was 828.86 m^2^/g resulting in significant improvement compared to raw activated carbon; it is attributed to the chemical/thermal modification treatment that enhanced surface accessibility and porosity. The total pore volume was found to be 0.6576 cm^3^ /g, and the average pore diameter was approximately 6.5 nm, indicating a mesoporous nature suitable for heavy metal adsorption^46^. The nanopore volume and nanopore specific area for MAC are 0.2815 cm^3^/g and 138.82 m^2^/g, respectively. The pore size distribution plot derived from the BJH method (Fig. 3(b)) further supports the mesoporous character of MAC, showing a sharp peak centered around 10–15 nm, which corresponds to the dominant pore width range. Additional smaller contributions from larger pores (> 50 nm) suggest the existence of macrospores that may facilitate faster diffusion of Ni (II) ions during adsorption.

These textural characteristics confirm that the modified activated carbon possesses a hierarchical pore structure, combining both meso- and macroporosity, which is highly beneficial for the removal of nickel ions from aqueous solutions. The enhanced surface area and tailored porosity promote efficient metal ion transport, accessibility to active sites, and overall high adsorption capacity.

**Fig. 4:**
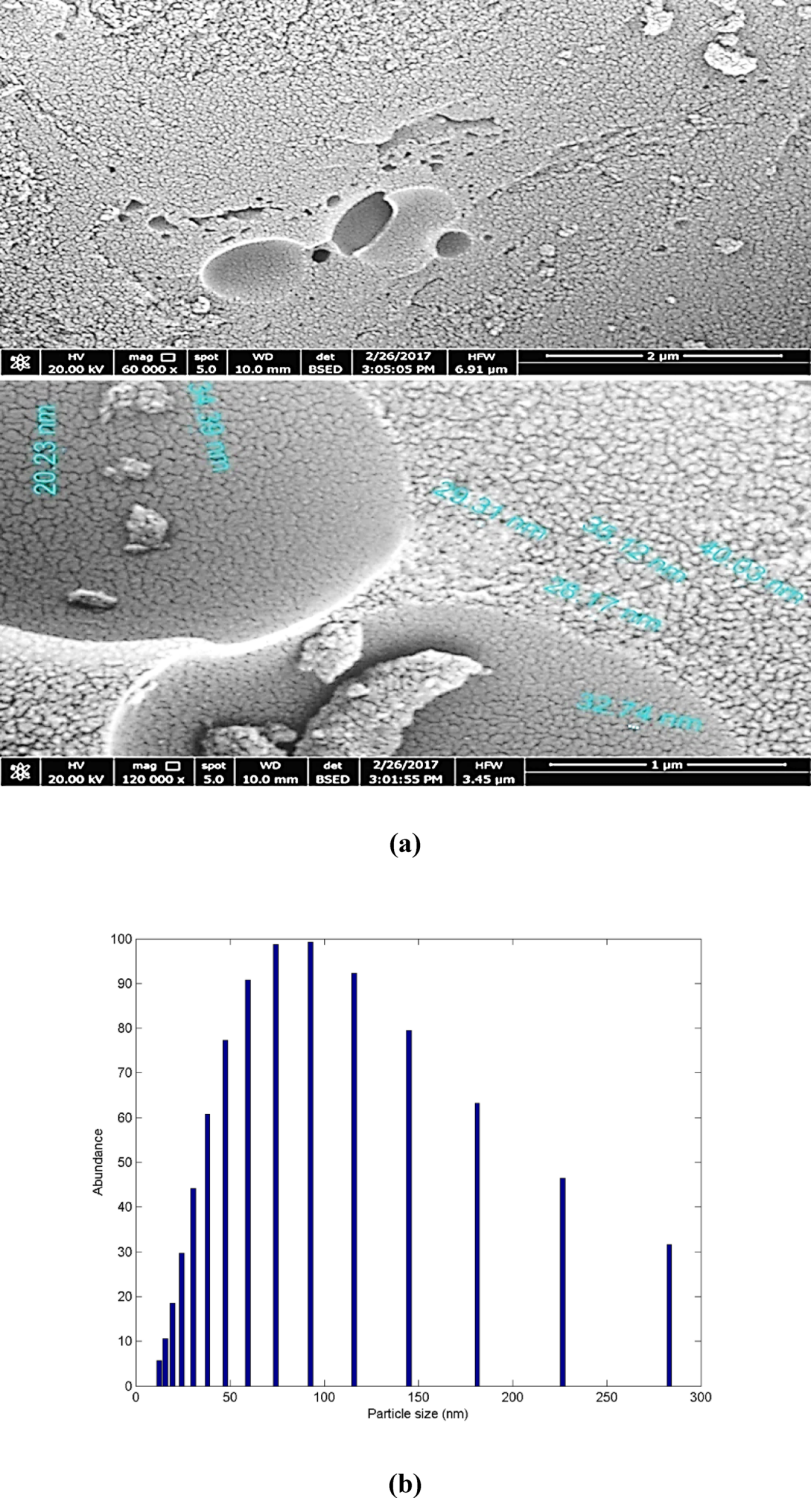
(**a**) High resolution scanning electron microscope (HRSEM) image of the MAC, (**b**) Particle distribution of the MAC.

The surface morphology of the modified activated carbon (MAC) was examined using Scanning Electron Microscopy (SEM), as depicted in Fig. 4a. The image reveals a highly heterogeneous and porous surface structure, which is a key attribute for efficient adsorption performance. The micrograph exhibits spherical and semi-spherical agglomerates with well-defined granular textures, indicating the successful activation and structural modification of the carbon matrix. The observed surface is densely covered with micro- and mesopores, enhancing the specific surface area and providing abundant active sites for adsorbate interaction. The granular surface texture and the porous nature are consistent with typical activated carbon materials but appear more pronounced due to the modification process employed^47^.

Particle size analysis from the SEM image indicates that the MAC consists of aggregated nanoscale features with diameters ranging approximately from 50 to 150 nm (Fig. 4(b)). These values confirm the nanoscale structure of the activated particles, which contributes to the increased surface-to-volume ratio and enhanced accessibility to the internal pore structure. The presence of cracks and fine pores across the surface suggests the formation of a highly porous architecture, most likely resulting from chemical and/or thermal activation treatments. Such features are advantageous for adsorption processes, particularly for metal ions like Ni (II), as they facilitate rapid diffusion and binding within the porous network.

### Adsorption optimization for Ni (II) ions

The central composite design (CCD) of 54 adsorption of Ni (II) ions on MAC surface trials was conducted to verify the contribution of different major factors; time, pH, MAC dose, and initial Ni (II) ions concentration on the adsorption process. CCD matrix for the measured and the calculated results of the removal capacity are listed in Table 1. It is noticed that the adsorption capacities of Ni (II) ions showed a strongly variant values with the variation of the independent factors. The multiple regression analysis of the obtained data confers the following second order polynomial equation, which exemplifies the relation between the Ni (II) adsorption capacity (response, y) and the input factors, time (x_1_), pH(x_2_), dose (x_3_) and initial concentration (x_4_):

$$\begin{aligned} y = & 163.2 - 0.07x_{1} - 9.2x_{2} - 255.3x_{3} + 0.21x_{4} - 0.022x_{1} x_{2} - 0.0036x_{1} x_{3} \\ & + 0.003x_{1} x_{4} - 6.1x_{2} x_{3} - 0.0195x_{2} x_{4} - 0.21x_{3} x_{4} \\ & + 0.0002x_{1}^{2} + 1.92x_{2}^{2} + 141.2x_{3}^{2} + 0.002x_{4}^{2} \\ \end{aligned}$$

**Fig. 5:**
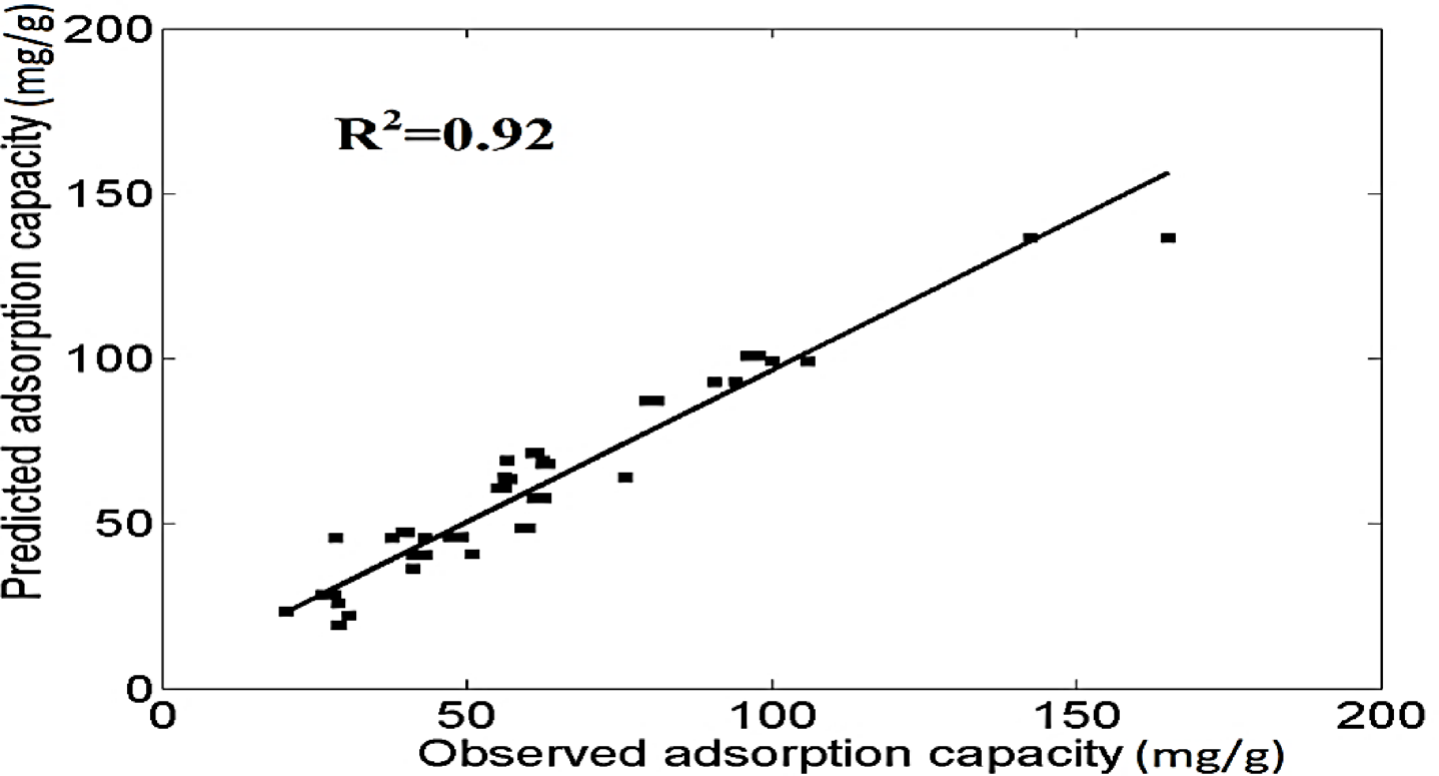
Plot of the observed versus the predicted values of Ni (II) removal capacity.

The statistical significance of the model is verified using analysis of variance (ANOVA) technique. The *P*-value (probability) of the model terms is calculated at 95% confidence level. The results from ANOVA test for the removal capacity of Ni (II) ions are given in Table 2. From the analysis of variance, the calculated F-value (F = 31.81) and the extremely low *P*-value (*P* < 0.0001) assure a highly significant model. Figure 5 illustrates the relation between the experimental removal capacity values and the predicted ones. There is a good conformity between the experimental and the predicted removal values with R^2^ value of 0.92. The value of R^2^ indicates that 92% of the variations of the response are due to the model variables that assures adequate adjustment between the number of experimental runs and the model variables. The model significance was assessed by calculating the t- values and *P*-values for each coefficient as in Table 3. The results showed that dose has a highly significant effect on adsorption of nickel ion on MAC surface with *P*-value (*P* < 0.0001) and high t- value but other variables have insignificant effect on adsorption process. Therefore, only MAC dose has the highest effect on the removal capacity.

**Table 2:** ANOVA test for the regression model.

Source of variance	degree of freedom	Sum of squares	Mean square	F-value	*P*-value
Regression	14.00	40,823.68	2915.97	31.81	0.0001
Residual	39.00	3574.97	91.6660	**–**	**–**
Lack of fit	10	3060.393	306.039	17.247	0.0001
Pure error	29	514.582	17.744	**–**	**–**
Total	53.00	44,398.66	**–**	**–**	**–**

**Table 3:** Calculated parameters of the quadratic polynomial coefficients.

Coefficient	calculated-value	t-value	*P*-value
${\beta }_{0}$	163.17	3.4444	0.0014
${\beta }_{1}$	− 0.0680	− 0.1666	0.8686
${\beta }_{2}$	− 9.1539	− 1.1244	0.2677
${\beta }_{3}$	− 255.3305	− 6.4797	0.0001
${\beta }_{4}$	0.2146	0.4378	0.039
${\beta }_{12}$	− 0.0218	− 0.5890	0.5593
${\beta }_{13}$	− 0.0036	− 0.0194	0.9846
${\beta }_{14}$	0.0030	1.3469	0.1858
${\beta }_{23}$	− 6.0914	− 1.6493	0.1071
${\beta }_{24}$	− 0.0195	− 0.4390	0.6631
${\beta }_{34}$	− 0.2069	− 0.9167	0.3649
${\beta }_{11}$	0.0002	0.1027	0.9187
${\beta }_{22}$	1.9269	2.7221	0.0096
${\beta }_{33}$	141.2066	8.7841	0.0001
${\beta }_{44}$	0.0020	8.7841	0.4026

### Response surface methodology of adsorption activity

**Fig. 6:**
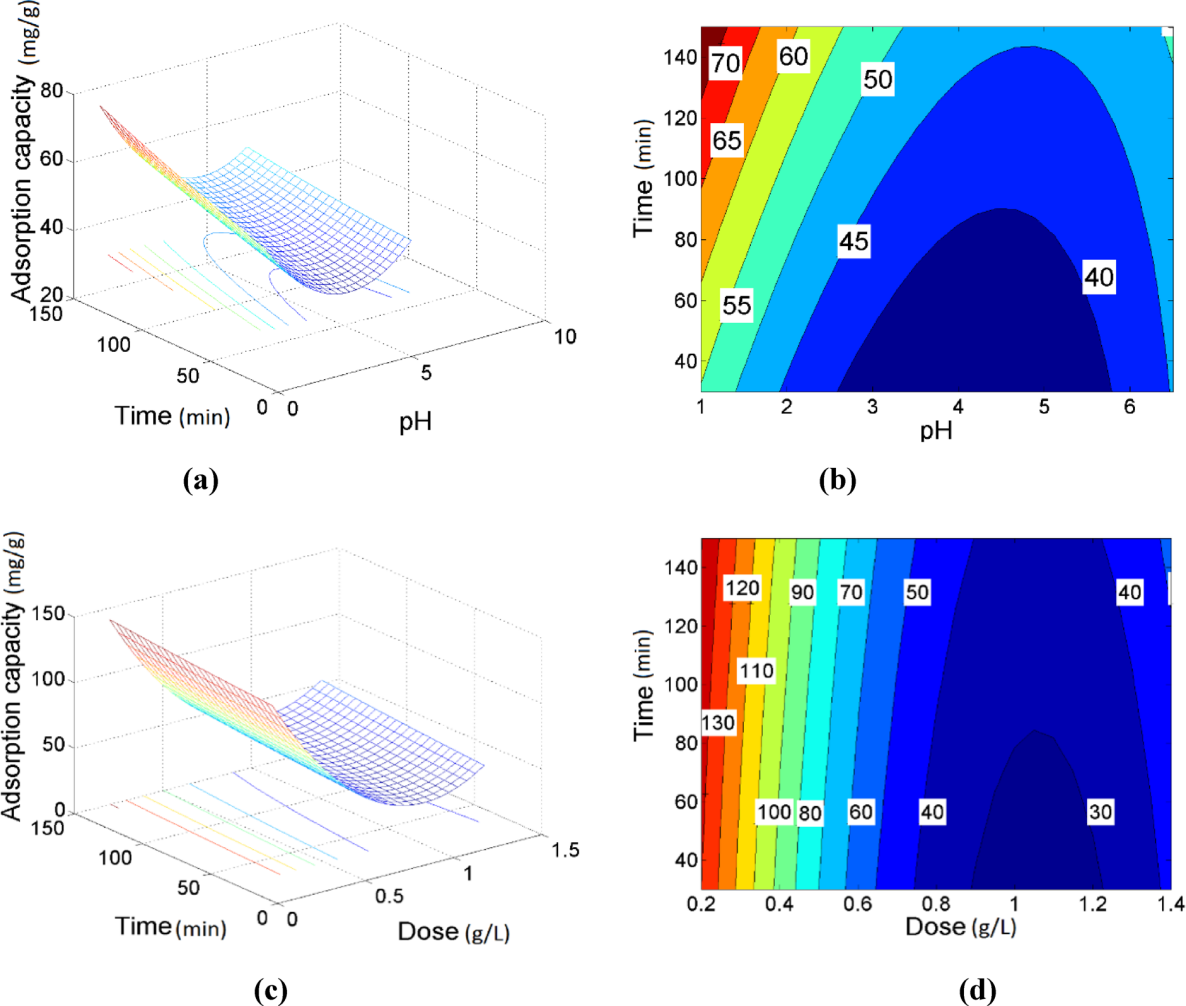
Response surfaces of predicted nickel removal (**a**, **c**) and the related contours (**b**, **d**) subjected to the effect of time and pH, time and dose, respectively, at the central points of other variables.

Three-dimensional response surface methodology (RSM) plots and the corresponding contour diagrams were utilized to investigate the influence of process variables on the adsorption of Ni (II) ions by modified activated carbon (MAC). Figure 6(a) illustrates the combined effect of contact time and solution pH on the adsorption capacity at a fixed adsorbent dose of 0.8 g/L and an initial Ni (II) ion concentration of 75 mg/L. The results indicate that Ni (II) ion uptake increased with decreasing pH and increasing contact time. However, the interaction between pH and contact time exhibited a minimal impact on enhancing the adsorption capacity.

Furthermore, the simultaneous increase in both contact time and adsorbent dose resulted in a slight reduction in removal efficiency, possibly due to site saturation or particle aggregation at higher doses. The contour plot in Fig. 6(b) provides a clearer quantitative estimation of the maximum adsorption capacity, with the highest value of approximately 74 mg/g achieved at pH 1 and a contact time of 150 min. The elliptical shape of the contour plot suggests a significant interaction between contact time and low pH conditions, indicating that pH is a critical factor in optimizing Ni (II) adsorption. These observations are further supported by FTIR analysis (see Fig. 2), which confirms the involvement of functional groups such as carboxylic and hydroxyl groups in the adsorption process. These groups become more protonated and reactive at lower pH, facilitating stronger chemical interactions between the MAC surface and Ni (II) ions.

Figure 6(c), d illustrate the interactive effect of contact time and adsorbent dose on the adsorption capacity of modified activated carbon (MAC) for Ni (II) ions. As shown in Fig. 6(c), the adsorption capacity decreases with increasing MAC dosage. The adsorption efficiency was found to be lowest within the dose range of 0.8–1.4 g/L, suggesting that higher adsorbent concentrations may lead to aggregation of particles or reduced effective surface area, thereby limiting adsorption performance. As well, variations in contact time exhibited a minimal influence on the adsorption capacity within the tested dose range, indicating that the dose has a more dominant effect under these conditions. The maximum adsorption capacity of 130 mg/g was achieved at a contact time of 150 min and an adsorbent dose of 0.24 g/L, as evidenced by the contour plot in Fig. 6(d).

**Fig. 7:**
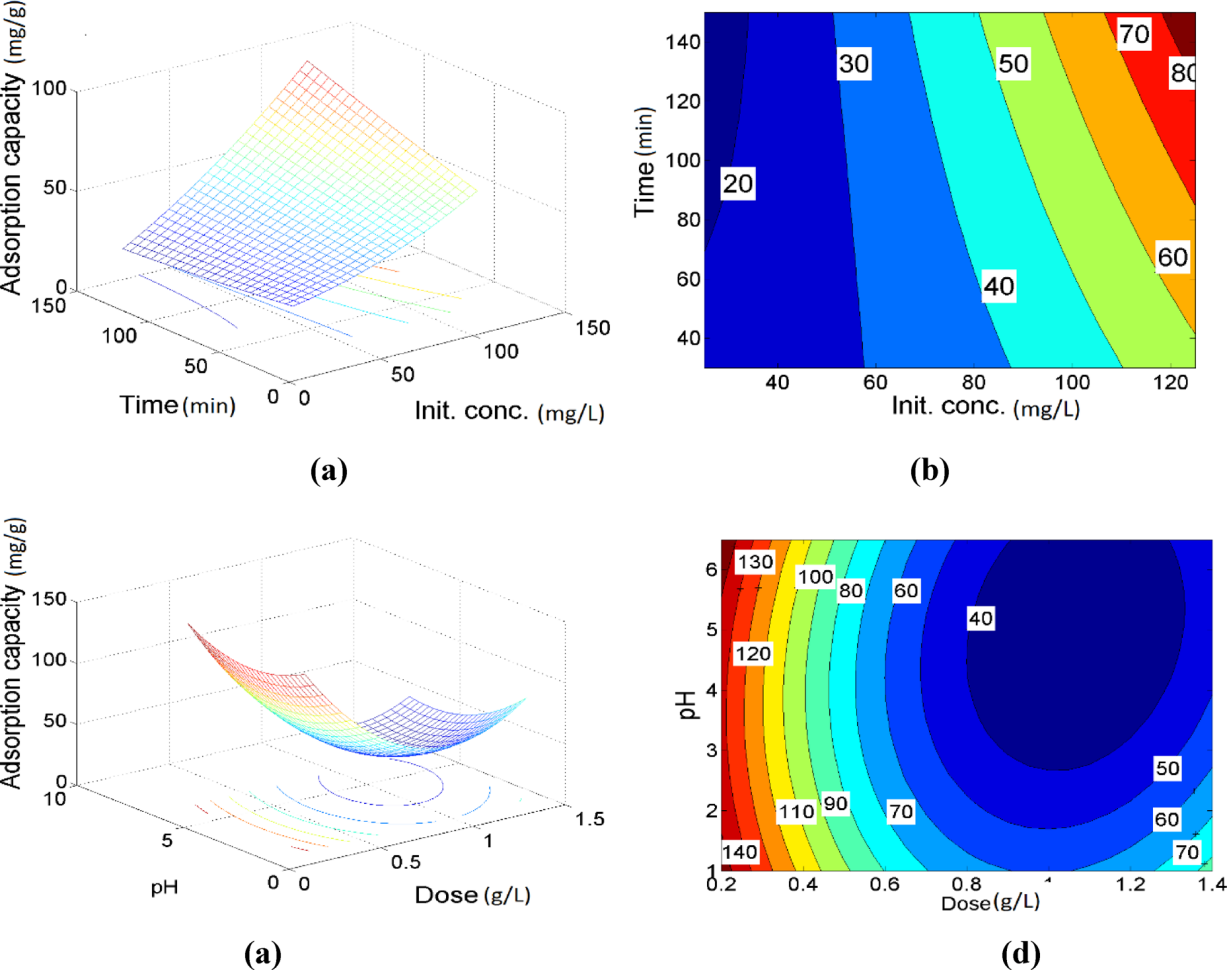
The 3D plots of the predicted nickel removal (**a**, **c**) and the related contour plots (**b**, **d**) under the effect of time-initial concentration, pH-dose, respectively.

**Fig. 8:**
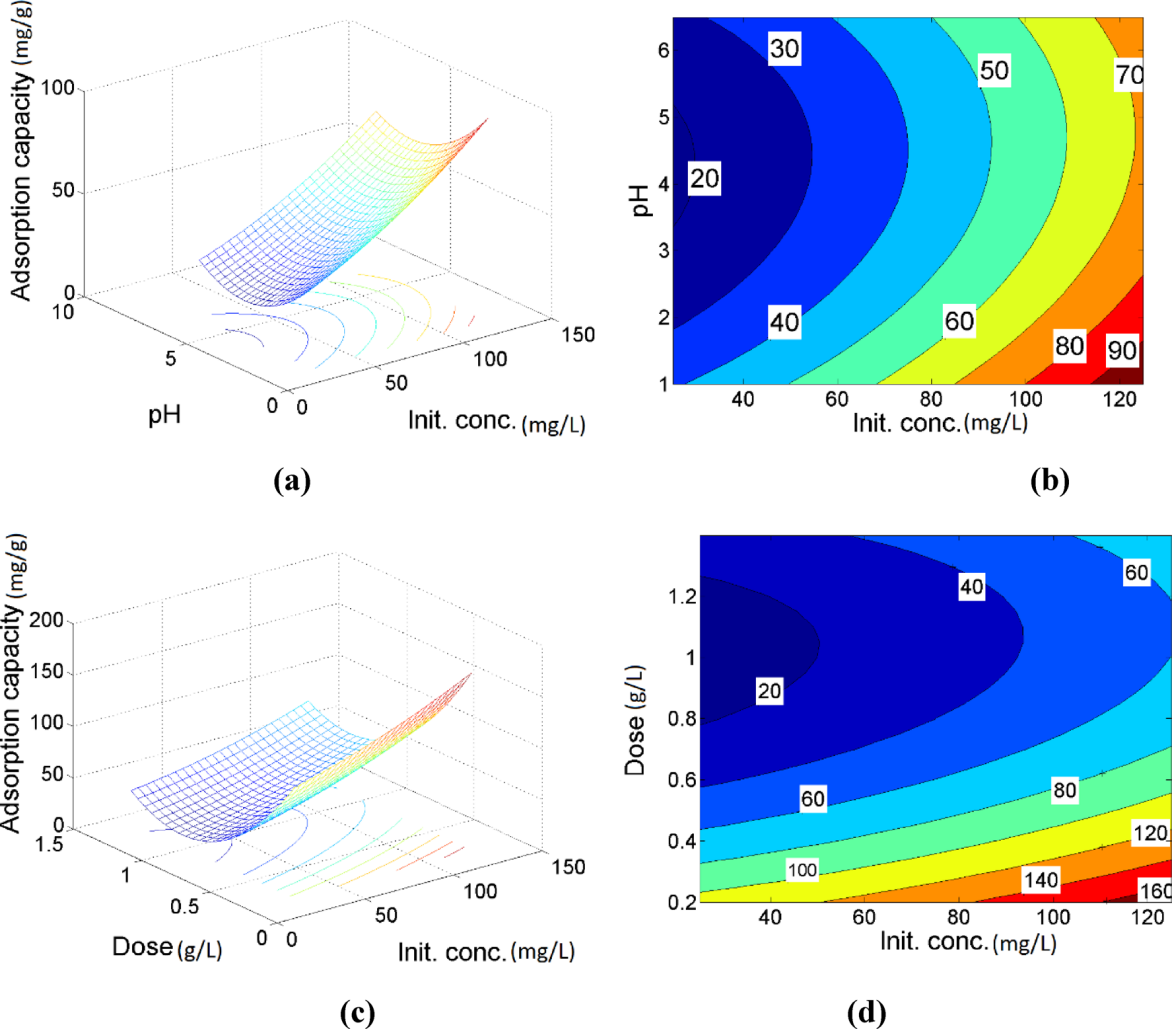
The 3D plots of the predicted nickel removal (**a**, **c**) and the related contour plots (**b**, **d**) as a function of pH-initial concentration, dose-initial concentration at the central values of other variables.

Generally, the response surface and contour analyses indicate that the combined interaction between contact time and adsorbent dose has a statistically insignificant effect on the adsorption of Ni (II) ions by MAC, with individual parameters playing a more critical role in influencing the process. The adsorption behavior of Ni (II) ions onto MAC as a function of the combined influence of contact time and initial ion concentration is illustrated in Fig. 7(a, b). The results indicate an approximately linear trend in the removal rate, suggesting that the interaction between time and initial concentration has an insignificant effect on Ni (II) adsorption. As shown in Fig. 7(c), the adsorption capacity of Ni (II) ions by MAC decreases with increasing adsorbent dose across various pH levels. A lower MAC dose results in a higher adsorption capacity, likely due to the greater availability of active sites per unit mass. In contrast, higher doses may lead to particle agglomeration, thereby reducing the effective surface area and number of accessible active sites for adsorption^48,49^. Furthermore, Fig. 7(d) demonstrates a significant interaction between pH and adsorbent dose during the adsorption process. The minimum removal capacity was observed at a pH of 5 and a dose of 1 g/L. The elliptical contour lines in Fig. 6(d) indicate a strong interaction between initial Ni (II) concentration and contact time, emphasizing their combined effect on the adsorption efficiency. Figure 8(a, b) demonstrates the combined effects of pH and initial Ni (II) ion concentration on the adsorption capacity of MAC. As shown in Fig. 8a, the adsorption capacity increases with higher initial Ni (II) concentrations and lower pH values. Elevated initial concentrations enhance the mass transfer rate of Ni (II) ions from the bulk solution to the MAC surface, thereby promoting greater interaction between the metal ions and the adsorbent^50^. A moderate interactive effect between pH and initial Ni (II) concentration is observed, as evidenced by the contour plot in Fig. 8(b). Furthermore, Fig. 8(c) reveals that higher initial concentrations coupled with lower MAC doses lead to an increase in Ni (II) adsorption capacity. This interaction between MAC dose and initial Ni (II) concentration is statistically significant, as confirmed by the contour plot in Fig. 7(d). At lower adsorbent doses, particle agglomeration is minimized, which helps maintain a greater number of accessible active sites for adsorption, thereby enhancing overall adsorption efficiency.

### Global effect on the removal capacity

**Fig. 9:**
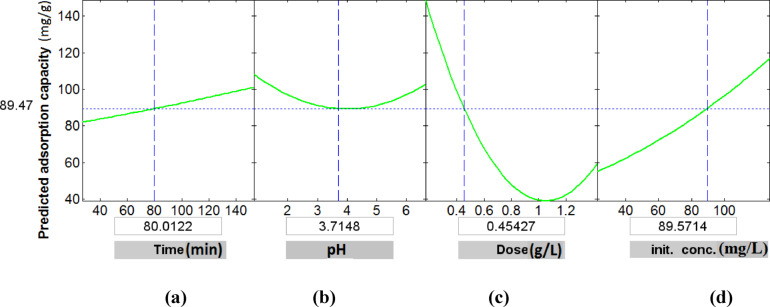
GUI for the variations in the modeled nickel removal capacity of Ni (II) ions with all variables.

Graphical User Interface (GUI) analysis was employed to optimize the combined effects of key operational parameters on the adsorption capacity of Ni (II) ions by MAC. Fig. 9 presents the influence of individual variables—contact time, pH, adsorbent dose, and initial Ni (II) ion concentration—on the adsorption capacity. Each plot demonstrates the predicted response variation with a single independent variable while maintaining all other variables constant, as indicated in the boxes beneath each axis. Altering the value of one independent variable adjusts the predicted response across the remaining conditions to reflect its specific influence. The optimal adsorption capacity was determined within the experimental design range: contact time (30–150 min), pH (1–6.5), adsorbent dose (0.2–1.4 g/L), and initial Ni (II) concentration (25–125 mg/L). Among these, the adsorbent dose exhibited the most pronounced effect on the removal capacity, followed by the initial metal ion concentration. These findings are consistent with the statistical significance indicated by the *P*- and *t*-values in Table 3.

Based on GUI analysis, the maximum predicted adsorption capacity of Ni (II) ions was estimated to be 206.26 mg/g under optimal conditions: contact time of 150 min, pH 1, MAC dose of 0.2 g/L, and initial Ni (II) concentration of 125 mg/L. To validate the predictive accuracy of the response surface model (RSM), an additional experiment was conducted under the same optimal conditions. The experimentally obtained adsorption capacity was 210 mg/g, closely matching the predicted value and thereby confirming the reliability and precision of the developed model.

### Adsorption kinetics

**Fig. 10:**
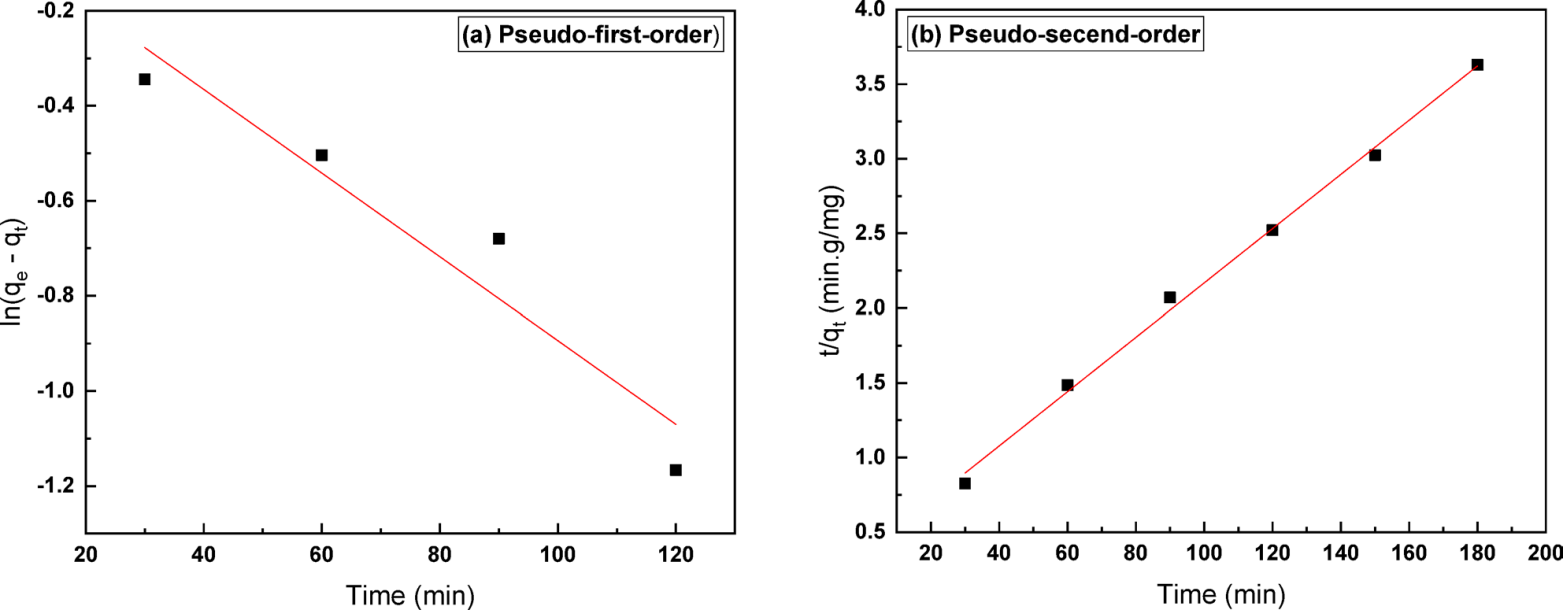
Kinetic plots for Ni (II) adsorption onto MAC, (**a**) pseudo-first-order linear plot, (**b**) pseudo-second-order linear plot.

Kinetic analysis was performed by fitting the experimental uptake data to both pseudo-first-order and pseudo-second-order models. The pseudo-first-order fit yielded R^2^ = 0.9184 ((Fig. 10(a)), while the pseudo-second-order fit gave R^2^ = 0.9968 (Fig. 10(b)), indicating a substantially better agreement with the PSO model. The close agreement of the PSO model with experimental data suggests that the rate of Ni (II) uptake on MAC is likely controlled by chemisorption involving electron sharing or exchange with surface functional groups. The kinetic parameters (k₁, k_2_) and fitting statistics are summarized in Table 4.

**Table 4:** Kinetic parameters for Ni (II) adsorption onto MAC.

Model	Parameter	Value
Pseudo-first-order	k_1_​ (min⁻^1^)	0.0088
	R^2^	0.9184
Pseudo-second-order	k_2_ (g mg⁻^1^ min⁻^1^)	9.46 × 10⁻^4^
	R^2^	0.9968

### Adsorption isotherm modeling

To gain deeper insight into the adsorption behavior of Ni (II) ions onto the MAC adsorbent surface, three well-established isotherm models were applied: Langmuir, Freundlich, and Dubinin–Radushkevich (D–R). These models are commonly used to elucidate the nature of adsorption processes, whether they are monolayer, multilayer, or involve chemisorption or physisorption mechanisms. The nonlinear Langmuir isotherm assumes monolayer adsorption onto a homogeneous surface containing a finite number of identical active sites. Its nonlinear form is expressed as:

7 $$q_{e} = \frac{{q_{max} bC_{e} }}{{\left( {1 + bc_{e} } \right)}}$$

where *q*_*e*_ (mg/g) is the amount adsorbed at equilibrium, *Ce* (mg/L) is the equilibrium Ni ions concentration in aqueous solution, *K*_*L*_ (L^-1^) is the Langmuir constant which related to the adsorption energy where, *K*_*L*_ and *Q*_*max*_ are Langmuir constants related to sorption energy and sorption capacity, respectively (Langmuir, 1916) ^68^.

**Fig. 11:**
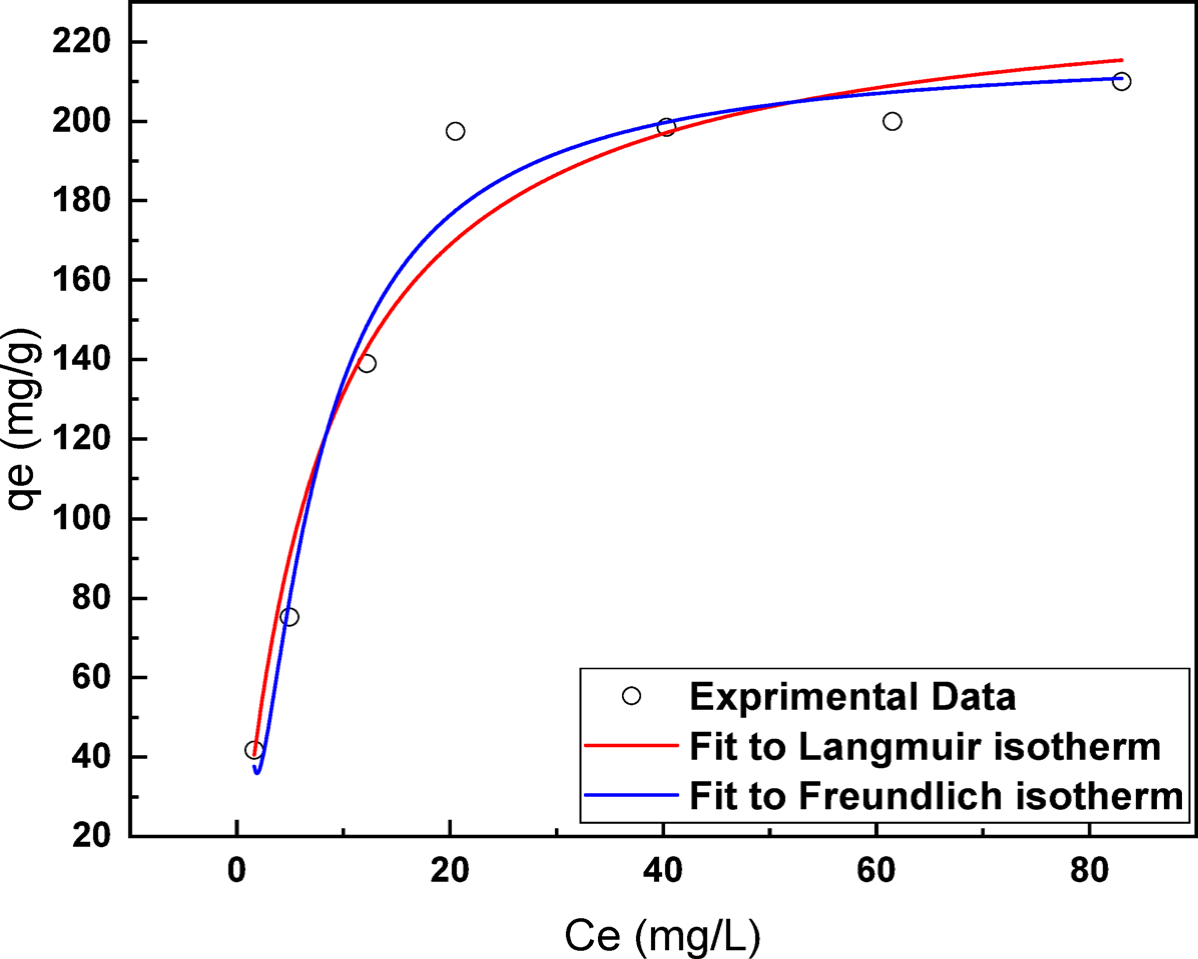
Adsorption isotherms (**a**) Langmuir adsorption isotherm, (**b**) Freundlich adsorption isotherm with nonlinear fittings (pH: 1, 0.2 g MAC/L, contact time: 150 min).

The nonlinear fitting of experimental data to the Langmuir model ((Fig. 11(a)) yielded a high regression coefficient (R^2^) of 0.996, confirming the suitability of this model in describing the adsorption mechanism achieving adsorption capacity of 236.5 mg/g. The calculated Langmuir separation factor, R_L_, which predicts the favorability of the adsorption process, ranged from 0.062 to 0.4002. As these values fall within the range 0 < R_L_ < 1, they indicate that the adsorption of Ni ions onto MAC is favorable across the tested concentration range.

The nonlinear Freundlich isotherm describes adsorption on heterogeneous surfaces and can be expressed by the following logarithmic form:

8 $${q}_{e}={K}_{f}{C}_{e}^\frac{1}{n}$$

where, *K*_*f*_ and *n* represent a Freundlich constants related to the adsorption capacity and intensity (Freundlich, 1907) ^67^.

As shown in Fig. 11(b), the Freundlich model also fit the data well, as evidenced by the high R^2^ value. The calculated constants were *K*_*f*_ value was 29.6 and *n* was greater than 1, indicating favorable adsorption and a strong interaction between Ni (II) ions and MAC. A value of n > 1 further supports the notion that the adsorption process is chemical in nature (chemisorption), rather than purely physical. The comparative fitting of experimental data to both Langmuir and Freundlich models demonstrates that the adsorption of Ni (II) ions onto MAC does not follow a purely monolayer mechanism, but instead suggests the presence of heterogeneous adsorption sites. This is consistent with the structural complexity and surface functionality of the MAC material, which provides a variety of active binding sites with different affinities. The calculated isotherm parameters are summarized in Table 4, confirming the strong affinity and high adsorption capacity of MAC for Ni (II) ions, attributed to surface complexation, electrostatic interactions, and possibly ion exchange processes. The consistency of both models with experimental data indicates that both monolayer and multilayer adsorption likely contribute to the overall mechanism.

**Fig. 12:**
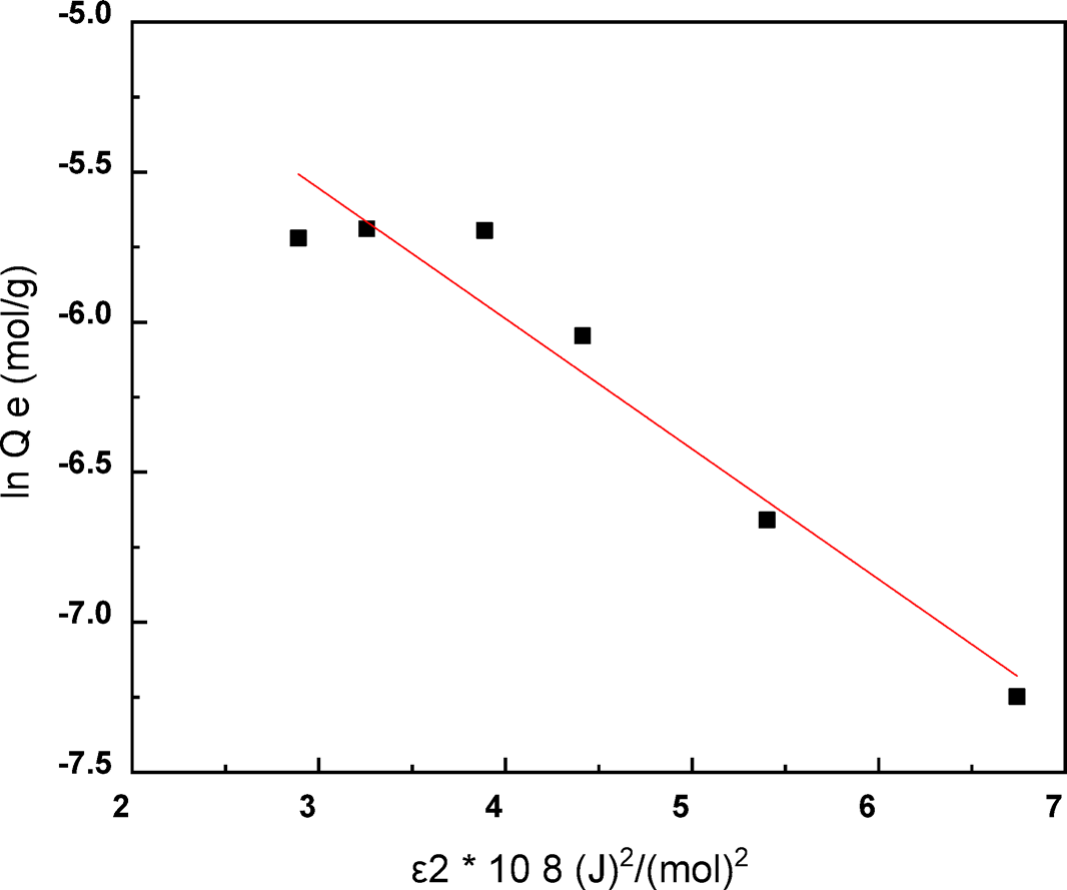
D–R adsorption isotherm model of Ni^2+^ ions adsorption on MAC.

To further investigate the adsorption mechanism of Ni (II) ions onto the MAC surface (Fig. 12), the Dubinin–Radushkevich (D–R) isotherm model was applied. Unlike the Langmuir and Freundlich models, which assume either homogeneous or heterogeneous adsorption surfaces, the D–R model provides insight into the nature of the adsorption process—particularly to distinguish between physical and chemical adsorption mechanisms.

The linear form of the D–R model is given by:

9 $$\ln q_{e} = \ln X_{m} - \beta \epsilon^{2}$$

where, *q*_*e*_ is adsorption capacity at equilibrium (mol/g), *X*_*m*_ is the theoretical D–R monolayer adsorption capacity (mol/g), *β* (mol^2^/J^2^) is a constant allied to adsorption energy, and *ε* (J/mol) is the Polanyi potential related to the equilibrium concentration, calculated using:

10 $$\epsilon = RT ln\left( {1 + {\raise0.7ex\hbox{$1$} \!\mathord{\left/ {\vphantom {1 {C_{e} }}}\right.\kern-0pt} \!\lower0.7ex\hbox{${C_{e} }$}}} \right)$$

In this equation, R is the universal gas constant (K = 8.314 J/mol), T is the absolute temperature (K), and C_e_ is the equilibrium concentration of Ni (II) ions in the solution (mol/L).

The mean adsorption energy E (kJ/mol), which provides critical information about the adsorption type, is calculated from the constant β as follows^37^:

11 $$E=\raisebox{1ex}{$1$}\!\left/ \!\raisebox{-1ex}{$\sqrt{2\beta }$}\right.$$

The magnitude of the mean free energy (E) of adsorption provides insight into the underlying adsorption mechanism. When the value of E is less than 8 kJ/mol, the process is primarily governed by physical adsorption, characterized by weak van der Waals forces between the adsorbate and the adsorbent surface. In the range of 8–16 kJ/mol, the adsorption mechanism is typically attributed to ion exchange or electrostatic interactions, indicating a semi-chemical nature involving either electrostatic attraction or partial sharing of electrons. When E exceeds 16 kJ/mol, the adsorption is considered to be chemical in nature, involving strong chemical bond formation between functional groups on the adsorbent and the adsorbate species. This classification helps in distinguishing between physical and chemical adsorption mechanisms and assessing the strength and type of interaction involved.

**Fig. 13:**
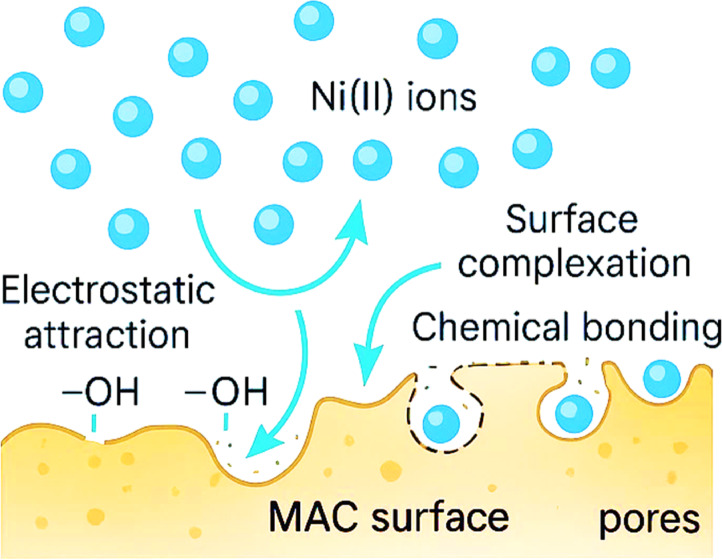
The schematic diagram that describe the adsorption of Ni ions on the MAC surface.

The (D–R) model parameters derived from the plot of ln q_e_ versus ε^2^ are summarized in Table 5. The linearity of the plot and the value of the correlation coefficient (R^2^) provide an indication of model fit. The calculated mean adsorption energy E was found to be 10.133 kJ/mol. Those results indicate the adsorption of Ni (II) onto MAC proceeds via chemisorption via ion exchange or electrostatic interactions with partial chemical characteristics. These findings are consistent with those obtained from Langmuir and Freundlich models and further confirm the nature of the interaction between Ni (II) ions and the surface-active sites on MAC as illustrated in Fig. 13.

**Table 5:** Calculated constants of the studied isothermal models for Ni^2+^ adsorption onto MAC.

Langmuir model			Freundlich model			Dubinin–Radushkevich (D–R)			
K L/mg	Q _max_ (mg/g)	R^2^	K_f_	n	R^2^	X m (mol/g)	(mol^2^/j^2^)	E,KJ/mol	R^2^
0.149	236.5	0.986	29.62	1.615	0.996	9.18 × 10^–5^	0.487 × 10^–8^	10.133	0.97

### Comparison between adsorption of Ni ions onto MAC and different sorbent

The maximal removal capacity q_max_ (mg/g) via adsorption is an inestimable characteristic which helps in industrial scale applications^51^. Previous studies using different adsorbents have been executed for adsorption of Ni (II) ions. The higher adsorptive capacity and dose of MAC for removal of Ni (II) ion was evaluated with previously used adsorbents and the value data are summarized in Table 6. The data obtained showed higher removal capacity of MAC with lower applied dose for adsorption of Ni (II) ions. Also, MAC has superior activity for sorption of metal ions than some highly modified and costly materials^51–54^**.**

**Table 6:** Comparison of adsorption capacity of Ni ion onto MAC with different used adsorbent.

Material	Dose used (g/L)	q_max_ (mg/g)	pH (optimal)	Temperature (°C)	References
Flower globular magnesium hydroxide	0.6	287	7	25	Jiang et al.^52^
Functionalized pineapple aerogels	1	49	6	30	Lim et al.^53^
Carbon aerogel	2	12.29	5	25	Fonseca-Correa et al.^54^
Functionalized titanium oxide magnetic nanoparticle	1	75.8	6	25	Mousavi et al.^55^
PVA/TEOS/TMPTMS hybrid membrane	1	10.5	6	25	Sahebjamee et al.^56^
Macro porous Ni^2^⁺-imprinted chitosan foam	1	69.93	6	30	Guo et al.^57^
Ion imprinting polymer coated magnetic/MMCNTs	2	19.86	6	25	He et al.^58^
Date seed derived biochar	–	19.54	5	25	Mahdi et al.^59^
Montmorillonite/graphene oxide nanocomposite	–	178	7	30	Neelaveni et al.^60^
Magnetic CoFe_2_O_4_-@(E)-N-(2-nitrobenzylidene)-2-(2-(2 nitrophenyl)imidazolidine-1-yl) ethaneamine (NBNPIEA)	0.36	151.5	5.7	25	Mehrabi an Dil^61^
Metakaolin based geopolymer (MKG)	3.2	42	7.25	25	Kara et al.^62^
Lemon peel biomass chemically modified with TiO_2_	5	15.5	6	25	Herrera-Barros et al.^63^
Multi-wall carbon nanotube functionalized by glycerol	0.2	115.8	7.2	55	Rahmati et al.^64^
Activated carbon derived from tamarind seed	1	39.25	5	30	Janthabut et al.^65^
MAC via pre-oxidized with iron/H_2_O_2_	0.2	236.5	7	25	Current study

### Recyclability studies

**Fig. 14:**
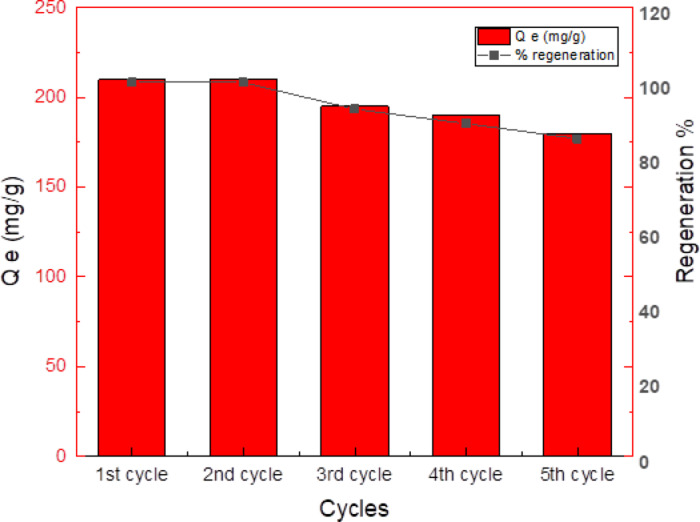
Regeneration performance of MAC over five adsorption–desorption cycles using 0.3 M HCl as the desorbing agent.

In view of environmental and economic sustainability, the reusability of the modified activated carbon (MAC) adsorbent was evaluated over five consecutive adsorption–desorption cycles. As shown in Fig. 14, both the adsorption capacity (Q_e_, mg/g) and regeneration efficiency (%) slightly decreased with each reuse cycle. Initially, the MAC exhibited a high adsorption capacity of approximately 210 mg/g in the first cycle, which gradually declined to around 180 mg/g by the fifth cycle. Similarly, the regeneration efficiency decreased from 100% to approximately 85% over the same period. This reduction can be attributed to gradual loss of surface functional groups and partial pore blockage caused by repeated exposure to acidic regenerants, which may slightly reduce the number of available active sites during re-adsorption.

Desorption of Ni(II) ions was effectively achieved using 0.3 M HCl, which enabled a regeneration efficiency of up to 85%, indicating the desorbing agent’s strong ability to disrupt interactions between the metal ions and the MAC surface. The slight reduction in performance across cycles can be attributed to potential structural changes in the MAC surface or partial blockage of active functional groups due to incomplete desorption. Overall, the MAC demonstrated excellent reusability with minor decline in performance, confirming its potential as a cost-effective and sustainable adsorbent for practical wastewater treatment applications.

The stability of MAC under highly acidic conditions was examined by exposing the adsorbent to pH 1 for 150 min, followed by washing with HCl and distilled water. The MAC retained its structural integrity after acid exposure, with no visible physical deterioration or loss of material, indicating good chemical resistance at extremely low pH.

### Cost analysis

To discover the economic and practical applicability of MAC, a cost analysis was executed. The cost estimation had mainly originated for MAC; chemicals cost and electricity consumption expenses as revealed by Table 7. All the costs and prices used in these calculations were estimated on 1 September, 2025. The expenditures for chemicals, and electricity, on unit scale were converted to US dollars ($) from Egyptian pound (EGP). The unit prices were multiplied by the quantity of the materials and electricity required to calculate the cost associated with each item. It is expected that the cost will still be reasonable even after considering the feedstock price.

**Table 7:** Materials input and costing for production of MAC.

Input	Amount required	Unit cost (L.E)*	Total cost	Cost ($)
Local Biochar (AC)	1 kg	30	30	0.6
Hydrogen perioxide	0.2 L	50	10	0.2
FeSO_4_	0.276 kg	10	2.76	0.055
H_2_SO_4_	0.2 L	7.2	1.44	0.029
Materials used cost	44.2	0.884		
Stirrer Energy consumed	1 kWh	2.3	2.3	0.046
Energy cost	2.3			
Total costing for 1 kg MAC (Materials used cost + Energy cost)	46.5	0.93		

*Prices are based on the Egyptian market during September 2025.

As shown in Table 7, for the production of 1 kgof MAC, biochar, hydrogen peroxide, sulfuric acid, and ferrous sulfate were required. Moreover, electricity consumed in stirring process was involved in cost estimation. The quantity of used chemicals, and electricity was calculated and presented in Table 7. The unit price of each item was calculated in US dollars ($) and the amount required was multiplied by the unit price. Net costs in materials used and electricity expenses were estimated and shown in the Table 7. For production of 1 kg of MAC, it was found material used expenses is ca 44.2 L.E (0.884 $). In the current study, for production of 1 kg of MAC, the cost was estimated and it was found that production of one kg of MAC is 46.5 L.E. (0.93 $) as shown in Table 7 that is lower than production cost of activated carbon which ranged from 1.82 to 2.15 $ per kg as previously reported^44,66^.

## Conclusion

In this work, a modified activated carbon (MAC) was effectively synthesized and employed for the adsorption of Ni (II) ions from aqueous solutions. The characterization of MAC using various analytical techniques confirmed its suitability as an efficient adsorbent. The FTIR spectra confirmed the presence of functional groups such as –OH, –COOH, and –C = O, which are crucial for binding metal ions. SEM imaging showed a highly porous surface morphology with abundant active sites. BET surface area analysis demonstrated that the high surface area and well-developed pore structure contributed significantly to the adsorption process.

Process optimization using a graphical user interface (GUI) approach highlighted the influence of contact time, pH, adsorbent dose, and initial Ni (II) concentration on adsorption efficiency. Under optimal conditions (150 min contact time, pH 1, 0.2 g/L dose, and 125 mg/L initial Ni (II) concentration), the predicted adsorption capacity was 206.26 mg/g, which was experimentally confirmed by a maximum uptake of 236.5 mg/g. The pseudo-second-order model provided an excellent fit with R^2^ = 0.9968, indicating that the adsorption process is better described by a chemisorption-controlled mechanism. Adsorption isotherm studies using Langmuir, Freundlich, and Dubinin–Radushkevich (D–R) models indicated that the process was favorable and occurred on a heterogeneous surface with chemisorption characteristics. The D–R model yielded mean adsorption energy (E) of 10 kJ/mol, indicating the predominance of a chemical adsorption mechanism.

Collectively, the structural features and surface chemistry of MAC, combined with its high adsorption capacity and favorable thermodynamic behavior, demonstrate that it is a promising, low-cost, and efficient material for the recyclability of Ni (II) from wastewater.

### Practical implications for industrial application

The optimized MAC adsorption system exhibits strong potential for practical deployment in real industrial wastewater treatment. Its preparation involves low-cost reagents and simple synthesis steps, which support feasibility for large-scale production. The high removal efficiency, rapid adsorption behavior, and acceptable regeneration performance across multiple cycles highlight the material’s operational robustness. Moreover, the ability of MAC to perform effectively under acidic conditions aligns well with typical effluents originating from electroplating, mining, battery manufacturing, and metal-finishing processes. Therefore, the findings of this study suggest that the MAC-based system can serve as a scalable, economical, and reliable solution for industrial Ni (II) remediation.

## Data Availability

The datasets generated and/or analyzed during the current study are not yet publicly available as they are being prepared for deposition but are available from the corresponding author on reasonable request.
